# New Bisabosquals from *Stachybotrys* sp. PH30583 Elicited on Solid Media

**DOI:** 10.3390/molecules23071577

**Published:** 2018-06-29

**Authors:** Bao-Hui Ruan, Shu-Quan Li, Xue-Qiong Yang, Ya-Bin Yang, Ya-Mei Wu, Li-Jiao Shi, Hai-Yue Yin, Hao Zhou, Zhong-Tao Ding

**Affiliations:** Functional Molecules Analysis and Biotransformation Key Laboratory of Universities in Yunnan Province, School of Chemical Science and Technology, Yunnan University, 2st Cuihu North Road, Kunming 650091, China; rbh20111883962@126.com (B.-H.R.); shuquanli@163.com (S.-Q.L.); yangxq@ynu.edu.cn (X.-Q.Y.); m18469110115@163.com (Y.-M.W.); 18387390596@163.com (L.-J.S.); 18487197973@163.com (H.-Y.Y.); zhouhaoa@163.com (H.Z.)

**Keywords:** *Stachybotrys* sp. PH30583, medium induction, triprenyl phenol, cytotoxicity, anticoagulant activity, chemotaxonomic marker

## Abstract

*Stachybotrys* sp. PH30583 cultured in liquid medium only led to one structure type of novel isochroman dimers. Using the one strain-many compounds strategy, the reinvestigation of the metabolites from *Stachybotrys* sp. PH30583 cultured in rice solid medium led to the isolation of four triprenyl phenols, including two new bisabosquals and two known phenylspirodrimanes. Nitrobisabosquals A and B (**1** and **2**) are the first case of pyrrolidone-bisabosquals reported in literature. Totally different compounds were isolated using rice solid medium, compared with those isolated using liquid medium, so that rice solid medium presents a key factor in the production of triprenyl phenols. Compound **1** exhibited cytotoxicity against tumor cells, A-549, HL-60, MCF-7 SMMC-7721, and SW480, as well as weak anticoagulant activity with activated partial thromboplastin time (APTT) of 32.1 ± 0.17 s (*p* < 0.05 vs. Con.) at a concentration of 5 mM. Triprenyl phenol metabolites could be used as chemotaxonomic markers for *Stachybotrys*.

## 1. Introduction

**Figure 1:**
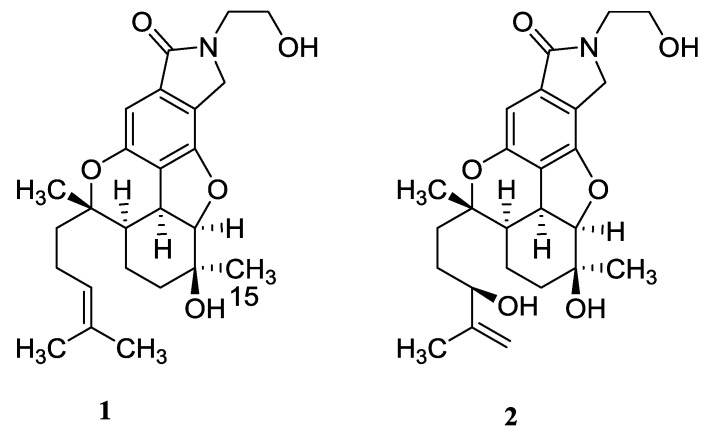
Structures of compounds **1** and **2**.

The genus *Stachybotrys* is an important source of active novel metabolites [1] that belong to the trichothecene [2], triprenyl phenol [3], diterpenoid [4], and isochroman [5] family of compounds. Some of these fungal metabolites have exhibited interesting bioactivities, such as antivirus [6], antibacterial [7], and cytotoxic activities [8]. The *Stachybotrys* species was reported to link to damp building–related illnesses [9]. As part of our continuous exploration for new metabolites from *Stachybotrys* sp. PH30583, we have previously reported a series of rare, novel isochroman dimers isolated from a liquid culture of the fungus [10]. We herein report the isolation and structure elucidation, on the basis of extensive spectral analysis, of two new bisabosquals, along with the in vitro evaluation of their cytotoxic and anticoagulant activity, and the isolation of two previously reported (reference) compounds (Figure 1), from a culture of the organism in solid medium. The bisabosquals, possessing a hexahydrobenzofurobenzopyran ring skeleton, represent a rare structural motif from the genus *Stachybotrys* [11]. Compounds **1** and **2** were the first example of pyrrolidone-bisabosqual in literature. Based on the structural analysis, the metabolites from *Stachybotrys* sp. PH30583, cultured separately in solid medium or liquid culture, were totally different. So, the cultivation mode of solid or liquid presents as the key factor in production of new compounds in *Stachybotrys* sp. PH30583.

## 2. Results and Discussion

**Figure 2:**
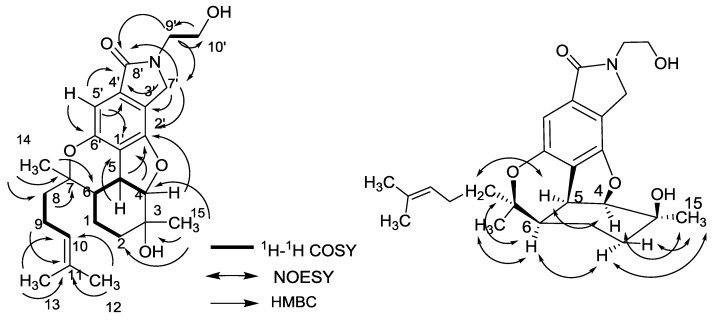
^1^H–^1^H correlation spectroscopy (COSY), heteronuclear multiple-bond correlation (HMBC) and nuclear Overhauser enhancement spectroscopy (NOESY) correlations of compound **1**.

**Figure 3:**
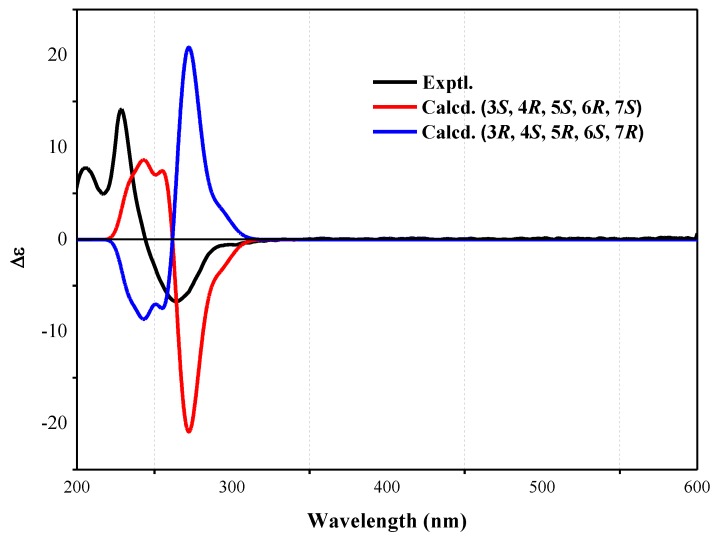
Experimental Electronic Circular Dichroism (ECD) (black) and calculated ECD spectra of (3*S*, 4*R*, 5*S,* 6*R*, 7*S*)-**1** (red) and (3*R*, 4*S*, 5*R*, 6*S*, 7*R*)-**1** (blue).

The molecular formula of nitrobisabosqual A (**1**) was determined to be C_25_H_33_NO_5_ on the basis of high resolution-electrospray ionization mass spectrum (HR-ESIMS) and ^13^C NMR data. The ^13^C and ^l^H NMR spectra of **1** revealed the presence of bisabosqual-type structure by comparing the NMR data with those for known bisabosquals A–D, whose structures were determined by X-Ray crystallographic analysis [11]. The difference between compound **1** and bisabosqual A was an additional pyrrolidone substituted in compound **1**, and it was the first example of pyrrolidone compound of the bisabosqual family in literature. The ^1^H–^1^H correlation spectroscopy (COSY) correlations between H-2/H-1/H-6/H-5/H-4; H-8/H-9/H-10; H-9′/H-10′, and the heteronuclear multiple-bond correlation (HMBC) correlations from H-5′, H-7′, and H-9′ to C-8′; H-5′ to C-1′ and C-4′; H-5 and H-7′ to C-2′; H-4 to C-1′; H-14 to C-6, C-7, and C-8; H-15 to C-2, C-3, and C-4; H-12 and H-13 to C-10, C-11; H-9 to C-11 also accorded with the structure of **1** (Figure 2). The relative configuration of **1** was determined by comparing the NMR data with those of bisabosqual A isolated from *Stachybotrys* [11]. It was also confirmed by the nuclear Overhauser enhancement spectroscopy (NOESY) correlations between H-4/H-5, H-4/H-15, H-5/H-8, H-6/H-8, H-15/H-2α, and H-2α/H-6. The absolute configuration of **1** was determined by quantum chemical Electronic Circular Dichroism (ECD) calculation. A pair of enantiomers ((3*S*, 4*R*, 5*S,* 6*R*, 7*S*)-**1** and (3*R*, 4*S*, 5*R*, 6*S*, 7*R*)-**1**) were used for the quantum chemical ECD calculation. The possible geometries were optimized by density functional theory (DFT) method at the B3LYP/6-31G(d,p) level, and the calculated ECD curve of (3*S*, 4*R*, 5*S,* 6*R*, 7*S*)-**1** was similar to the experimental one (Figure 3). Therefore, the absolute configuration of **1** was determined as 3*S*, 4*R*, 5*S,* 6*R*, 7*S*.

**Figure 4:**
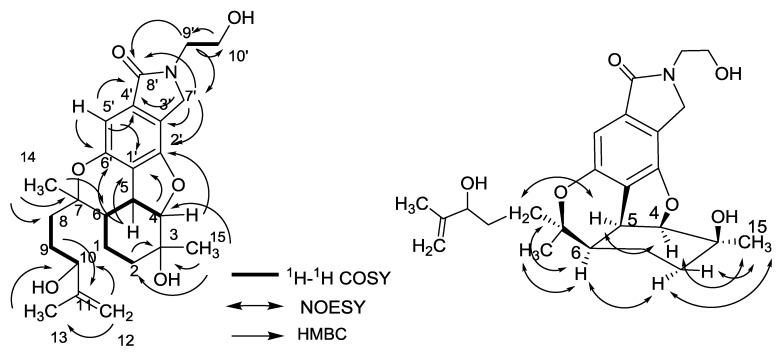
COSY, HMBC and NOESY correlations of compound **2**.

The molecular formula of nitrobisabosqual B (**2**) was determined to be C_25_H_33_NO_6_ by analyzing the HR-ESIMS and ^13^C NMR data. The ^13^C and ^l^H NMR spectra of **2** also indicated the existence of bisabosqual-type structure by comparing the NMR data with those of known bisabosquals A–D and compound **1**. The differences between the structures of compounds **1** and **2** were an OH at C-10 and a double bond at C-11 and C-12 in compound **2**. The COSY correlations between H-2/H-1/H-6/H-5/H-4; H-8/H-9/H-10; H-9′/H-10′, and HMBC correlations from H-5′, H-7′, and H-9′ to C-8′; H-5′ to C-1′ and C-6′; H-5 to C-1′ and C-6′; H-4 and H-7′ to C-2′; H-4 to C-1′; H-14 to C-6, C-7, and C-8; H-15 to C-2, C-3, and C-4; H-12 and H-13 to C-10; H-9 to C-11 also accorded with the structure of **2** (Figure 4). The relative configuration of **2** was determined by comparing the NMR with those of bisabosqual D isolated from *Stachybotrys* [11]. It was also confirmed by the NOESY correlations between H-4/H-5, H-4/H-15, H-5/H-8, H-6/H-8, H-15/H-2α, and H-2α/H-6. The absolute configuration of **2** was determined by comparing the Circular Dichroism (CD) spectrum (Supplementary Materials) with those of **1**.

Two known triprenyl phenols were also isolated and determined as stachybotrylactam and stachybotramide [12]. The *Stachybotrys* fungi are rich in triprenyl phenols, which contain stachybotrins, phenylspirodrimanes, bisabosquals, kampanols, and stachyflins [1]. Triprenyl phenols appear to be the characteristic compounds of *Stachybotrys*, indicating that this kind of metabolites could be used in the chemotaxonomy of this genus.

Metabolites **1** and **2** were evaluated for their cytotoxicity, anticoagulant, and anti-acetylcholinesterase activities. Compound **1** exhibited cytotoxicity against the cancer cell lines A-549, HL-60, MCF-7 SMMC-7721, and SW480, when tested at 40 μM demonstrating percent inhibitions of 14.22%, 47.41%, 49.02%, 20.14%, and 16.69%, respectively. However, compound **2** exhibited no obvious cytotoxicity. Compound **1** also exhibited weak anticoagulant activity with activated partial thromboplastin time (APTT) of 32.1 ± 0.17 s (*p* < 0.05 vs. Con.) at a concentration of 5 mM, while **2** exhibited no obvious anticoagulant activity with an APTT of 31.1 ± 0.30 s. Compounds **1** and **2** did not exhibit obvious anti-acetylcholinesterase activity with ratios of inhibition <10% at the concentrations of 50 μM. The structural dissimilarity between compounds **1** and **2** was the hydroxyl at C-10 in compound **2**, so the oxidation in **2** reduce the anticoagulant and cytotoxic activity in bisabosqual derivatives. 

## 3. Materials and Methods

### 3.1. General Experimental Procedures

Sephadex LH-20 (GE Healthcare Co., Buckinghamshire, UK), Silica gel (Qingdao MCG Co., Qingdao, China), and LiChroprep RP-18 (Merck, Darmstadt, Germany) were applied for column chromatography. One dimension and two dimension NMR spectra were acquired on a Bruker AVANCE 500 MHz and a Bruker 600 MHz NMR instrument (Bruker Co., Karlsruhe, Germany). MS spectra were obtained in an Agilent G3250AA system (Agilent, Santa Clara, CA, USA) and a Waters AutoSpec Premier P776 spectrometer (Waters, Milford, MA, USA). Optical rotations were acquired on a Jasco P-1020 polarimeter (Jasco Co., Tokyo, Japan). Circular dichroism spectra were obtained in an Applied Photophysics Chirascan spectrometer (Applied Photophysics Ltd., Surrey, UK).

### 3.2. Fungus and Fermentation

The fungus was isolated using potato dextrose agar medium (PDA) (potato 0.2 kg/L, agar 15 g/L, glucose 20 g/L) from soil sample in Tibetan and was authenticated as *Stachybotrys* sp. by ITS gene sequence (GenBank accession number KC305326). This fungus was conserved at Functional Molecules Analysis and Biotransformation Key Laboratory, Yunnan University, China. Fungus *Stachybotrys* sp. PH30583 was fermented in 0.5 L Erlenmeyer flasks each containing 0.2 L PDB seed fermentation broth (cut potato 0.2 kg/L, glucose 0.02 kg/L). After 3 days of culture at 28 °C with an incubator shaker (180 rpm), the seeds were cultured according to the amount of 10% cultured in rice. An amount of 40 kg rice medium was fermented at room temperature for 30 days. 

### 3.3. Extraction and Isolation of Compounds

All 40 kg of the fermented product was extracted with acetidin for three times, the acetidin extract was evaporated to obtain crude extract 250 g. The acetidin extract was separated into five fractions (fraction 1–fraction 5) with gradient elution (petroleum ether, chloroform/methanol 1:0, 30:1, 10:1, 3:1, *v/v*) on silica gel. Fraction 4 was separated by Sephadex LH-20 (methanol) (GE Healthcare Co, Buckinghamshire, UK) to afford the fraction 4.1–fraction 4.2. Fraction 4.1 was successively separated by Sephadex LH-20 (methanol) and silica gel column with acetidin /petroleum ether (1:1 *v/v*) to afford compound **1** (20 mg) and stachybotrylactam (36 mg). Fraction 4.2 was then subjected to further elution on LiChroprep RP-18 (Merck, Darmstadt, Germany) with step gradient elution (methanol/H_2_O, 10/90 to 100/0, *v/v*) and Sephadex LH-20 (MeOH) to give **2** (2 mg) and stachybotramide (3 mg). 

Nitrobisabosqual A (**1**): ${\left[\alpha \right]}_{D}^{23}$ = −47.0° (MeOH); ^1^H (MeOD, 600 MHz) and ^13^C NMR (MeOD, 150 MHz) data, see Table 1 and Supplementary Materials; HR-ESI-MS [M + H]^+^ *m/z* 428.2420 (calcd for C_25_H_34_NO_5_ 428.2437).

Nitrobisabosqual B (**2**): ${\left[\alpha \right]}_{D}^{23}$ = −34.5° (MeOH); ^1^H (MeOD, 500 MHz) and ^13^C NMR (MeOD, 125 MHz) data, see Table 1 and Supplementary Materials; HR-ESI-MS [M + H]^+^ *m/z* 444.2366 (calcd for C_25_H_34_NO_6_ 444.2386).

### 3.4. Bioactivity Assay

Acetylcholinesterase inhibitory activities of metabolites were performed by the spectrophotometric method according to Ellman et al. [13]. This method is applied for the evaluation of acetylcholinesterase inhibitory activities. The reagents were purchased from Sigma-Aldrich. The metabolites were dissolved in dimethyl sulfoxide. The 200 μL reaction mixture of phosphate buffer, the test sample with the concentration of 50 μM, and acetyl cholinesterase (0.02 U/mL), was incubated for twenty min at 37 °C. The latter reacted with 40 μL of 0.625 mM DTNB and 0.625 mM acetylthiocholine iodide solution. The absorbance was monitored at 405 nm every 30 s for one hour. A 0.333 μM tacrine solution was used as a positive control. All of the reactions were measured in triple. The percentage inhibition was calculated as the formula.

The in vitro anticoagulant activity of metabolites **1** and **2** were investigated by the method of APTT [14]. Low molecular weight heparin (LMWH) was used as positive control with APTT of 263.3 ± 1.65 s at a concentration of 35.6 μM. These samples were dissolved in dimethyl sulfoxide. In the APTT assay, plasma was blended with **1**, and **2** for two minutes at 37 °C. Then APTT assay reagent was added to the mix and incubated for three minutes at 37 °C. Finally, the coagulation time was recorded. 

The cytotoxicity of metabolites **1** and **2** against tumor cells, HL-60, A-549, SMMC-7721, SW480, and MCF-7 were assessed in vitro by the 3-(4,5-dimethylthiazol-2-yl)-5(3-carboxymethoxyphenyl)-2-(4-sulfopheny)-2H-tetrazolium (MTS) means [15]. The positive control of taxol was used with IC_50_ < 0.008 μM.

### 3.5. ECD Calculations

The theoretical calculations of **1** were performed using Gaussian Program [16] by Yunnan Electronic Computing Center. Two geometries of (3*S*, 4*R*, 5*S,* 6*R*, 7*S*)-**1** and (3R, 4S, 5R, 6S, 7*R*)-**1** were previously optimized by Density Functional Theory (DFT) methods at the B3LYP/6-31G(d,p) level and excitation energies and rotational strengths were calculated using time-dependent Density Functional Theory (TDDFT) at the B3LYP/6-31G(d,p) level, respectively. The ECD spectrum was simulated from electronic excitation energies and velocity rotational strengths.

## Figures and Tables

**Table 1:** ^1^H and ^13^C NMR spectral data of nitrobisabosquals A (**1**) and B (**2**).

Position	1		2	
	δ_C_ (ppm)	δ_H_ (ppm, *J* in Hz)	δ_C_ (ppm)	δ_H_ (ppm, *J* in Hz)
1	16.3	1.42, 1.15 (m)	16.2	1.53, 1.28 (m)
2	35.2	1.57, 1.15 (m)	35.1	1.69, 1.33 (m)
3	68.6		68.6	
4	91.8	4.74 (d, 8.4)	91.8	4.78 (brs)
5	34.4	3.57 (m)	34.3	3.77 (m)
6	36.2	2.04 (m)	35.9	2.11 (m)
7	81.4		81.3	
8	38.2	1.56, 1.48 (m)	35.1	1.70(m)
9	22.0	2.02 (m)	28.7	1.69 (m)
10	123.7	4.99 (m)	75.4	3.92 (m)
11	131.3		147.5	
12	24.4	1.57 (s)	110.3	4.91, 4.75 (m)
13	16.3	1.48 (s)	16.1	1.68 (s)
14	21.4	1.30 (s)	21.5	1.42 (s)
15	28.2	1.14 (s)	28.2	1.28 (s)
1′	115.3		115.3	
2′	155.9		156.0	
3′	134.5		134.4	
4′	113.6		113.6	
5′	101.6	6.57(s)	101.5	6.69 (s)
6′	151.7		151.6	
7′	47.6	4.53, 4.30 (d, 16.8)	48.1	4.66, 4.48 (d, 17.0)
8′	169.9		169.9	
9′	45.1	3.57(m)	45.0	3.73 (m)
10′	59.8	3.69 (m)	59.8	3.80 (m)

## Supplementary Materials

The following are available online. 1D, 2D NMR, MS and CD of the new compounds **1** and **2** (Figure S1–Figure S16).
