# Supplementary material for: New Bisabosquals from Stachybotrys sp. PH30583 Elicited on Solid Media
Source: Molecules. 2018 Jun 29;23(7):1577. doi: 10.3390/molecules23071577 (PMC6100399; doi:10.3390/molecules23071577)
Supplement: Supplementary file 1 [file molecules-23-01577-s001.pdf]

Supplementary Materials

New Bisabosquals from *Stachybotrys* sp. PH30583 Elicited on Solid Media

Bao-Hui Ruan †, Shu-Quan Li †, Xue-Qiong Yang, Ya-Bin Yang *, Ya-Mei Wu, Li-Jiao Shi, Hai-Yue Yin, Hao Zhou and Zhong-Tao Ding *

Functional Molecules Analysis and Biotransformation Key Laboratory of Universities in Yunnan Province, School of Chemical Science and Technology, Yunnan University, 2st Cuihu North Road, Kunming 650091, China; rbh20111883962@126.com (B.-H.R.); shuquanli@163.com (S.-Q.L.); yangxq@ynu.edu.cn (X.-Q.Y.); ybyang@ynu.edu.cn (Y.-B.Y.); m18469110115@163.com (Y.-M.W.); 18387390596@163.com (L.-J.S.); 18487197973@163.com (H.-Y.Y.); zhouhaoa@163.com (H.Z.); ztding@ynu.edu.cn (Z.-T.D.)

* Correspondence: ybyang@ynu.edu.cn (Y.-B.Y.); ztding@ynu.edu.cn (Z.-T.D.); Tel.: +86-871-650-33719 (Y.-B.Y.); +86-871-650-33910 (Z.-T.D.)

† These authors contribute equally to this work.

Table of contents

Figure S1. HRESIMS spectrum of compound **1** 3

Figure S2. ^{1}H NMR spectrum of compound **1** in MeOD (600 MHz) 3

Figure S3. ^{13}C NMR spectrum of compound **1** in MeOD (150 MHz) 4

Figure S4. COSY spectrum of compound **1** in MeOD (600 MHz) 4

Figure S5. HSQC spectrum of compound **1** in MeOD (600 MHz) 5

Figure S6. HMBC spectrum of compound **1** in MeOD (600 MHz) 5

Figure S7. ROESY spectrum of compound **1** in MeOD (600 MHz) 6

Figure S8. CD spectrum of compound **1** 6

Figure S9. HRESIMS spectrum of compound **2** 7

Figure S10. ^{1}H NMR spectrum of compound **2** in MeOD (500 MHz) 7

Figure S11. ^{13}C NMR spectrum of compound **2** in MeOD (125 MHz) 8

Figure S12. COSY spectrum of compound **2** in MeOD (600 MHz) 8

Figure S13. HSQC spectrum of compound **2** in MeOD (600 MHz) 9

Figure S14. HMBC spectrum of compound **2** in MeOD (600 MHz) 9

Figure S15. ROESY spectrum of compound **2** in MeOD (600 MHz) 10

Figure S16. CD spectrum of compound **2** 10

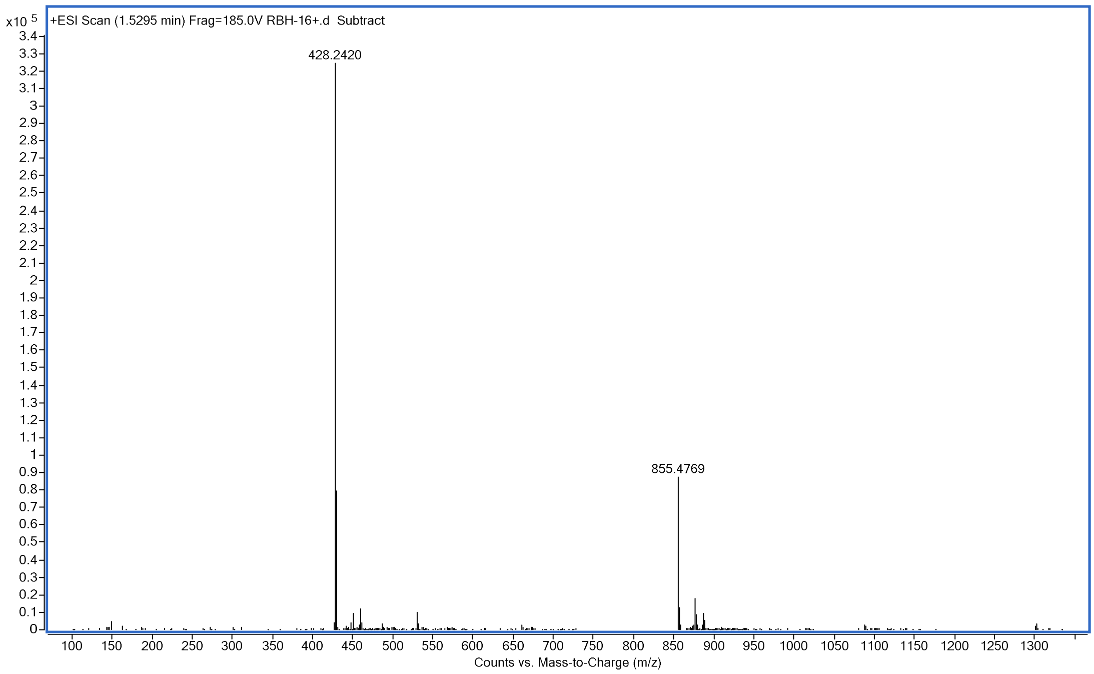

Figure S1. HRESIMS spectrum of compound **1**

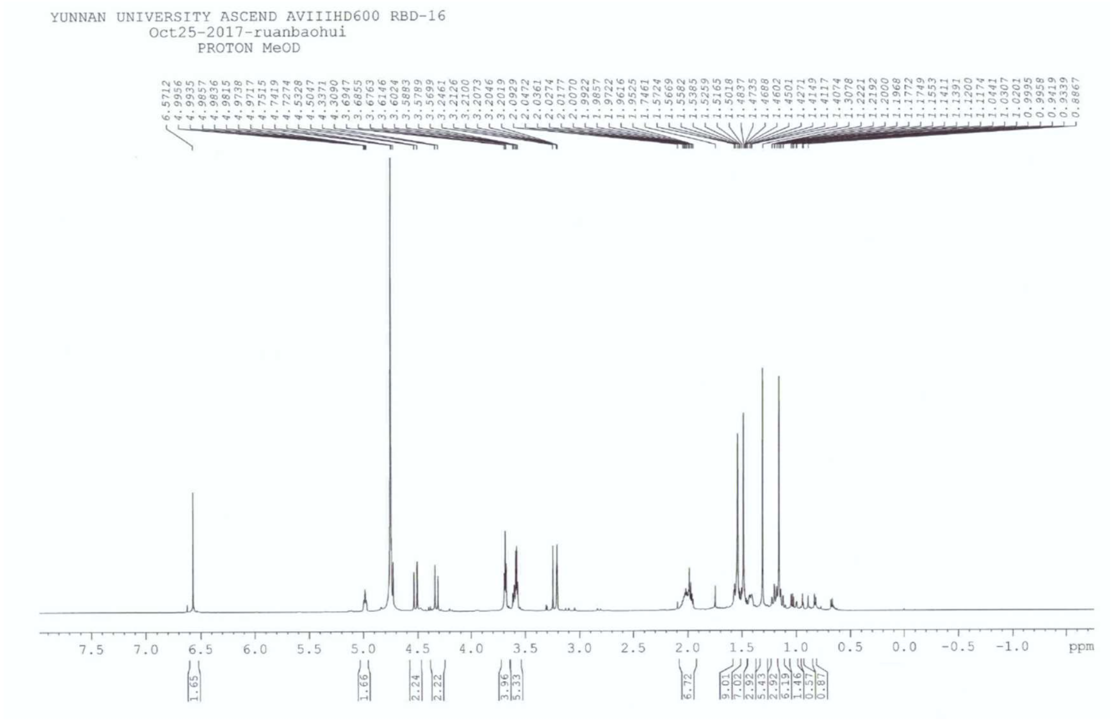

Figure S2. ^{1}H NMR spectrum of compound **1** in MeOD (600 MHz)

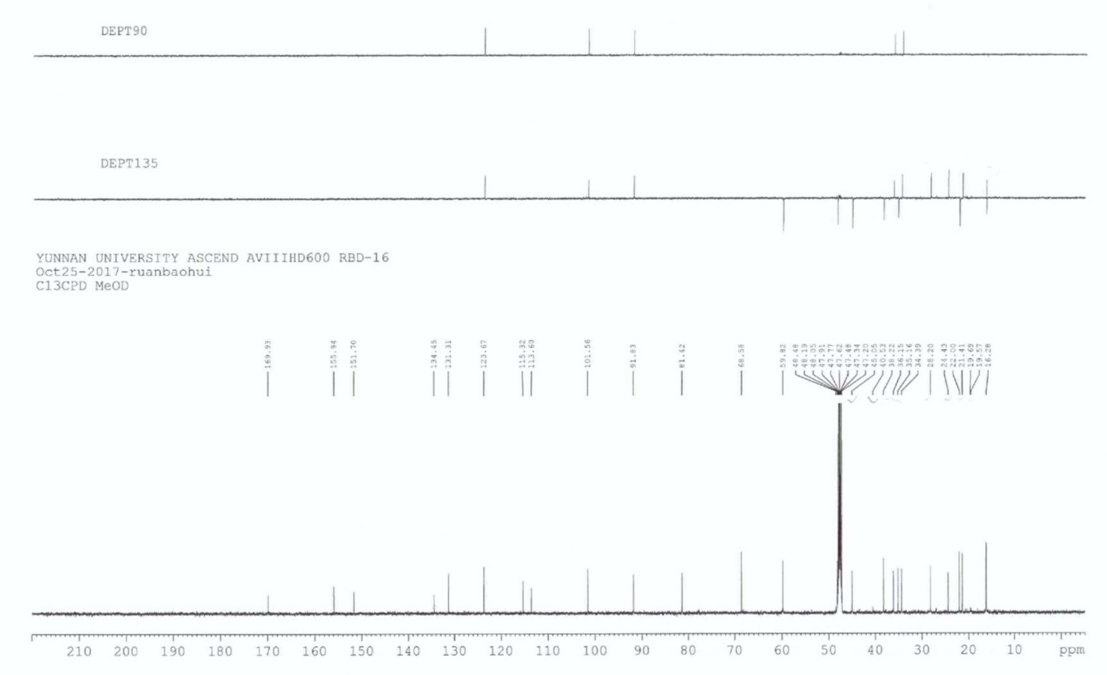

Figure S3. ^{13}C NMR spectrum of compound **1** in MeOD (150 MHz)

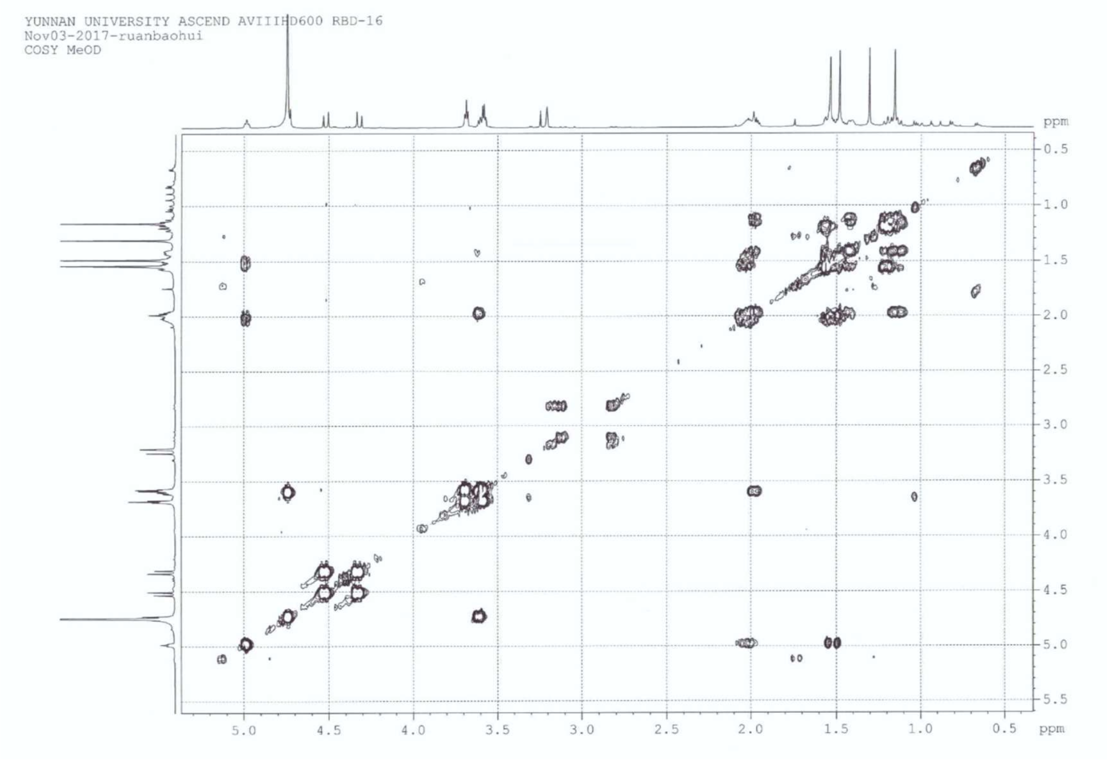

Figure S4. COSY spectrum of compound **1** in MeOD (600 MHz)

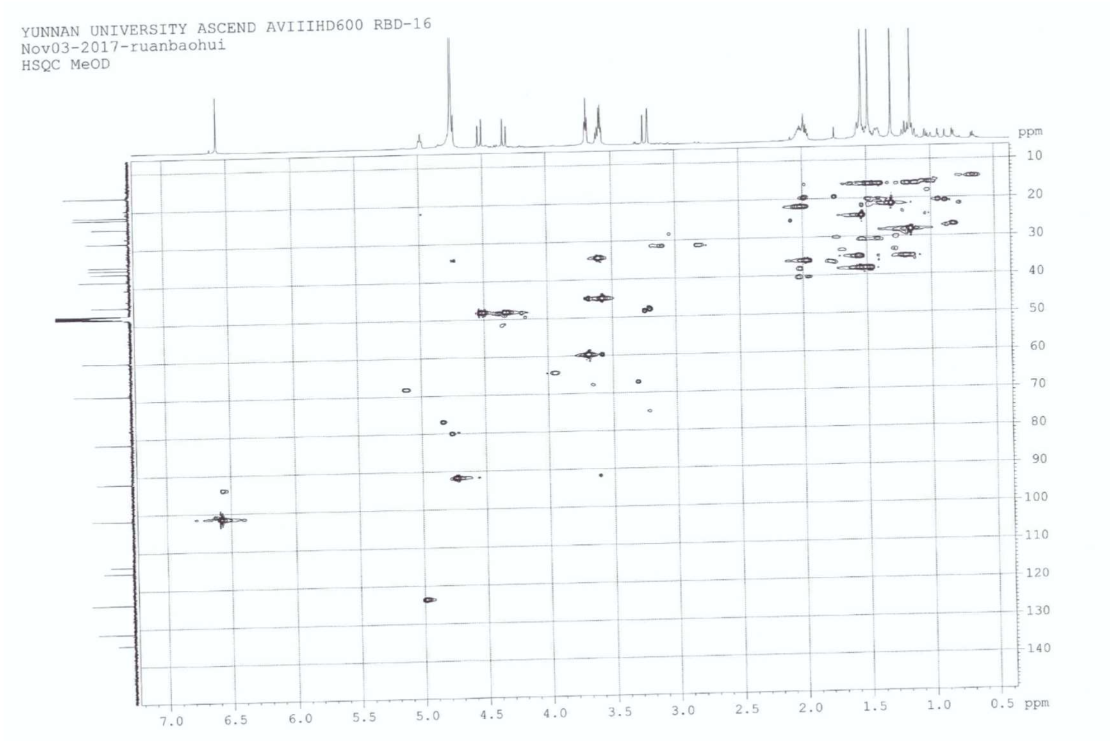

Figure S5. HSQC spectrum of compound **1** in MeOD (600 MHz)

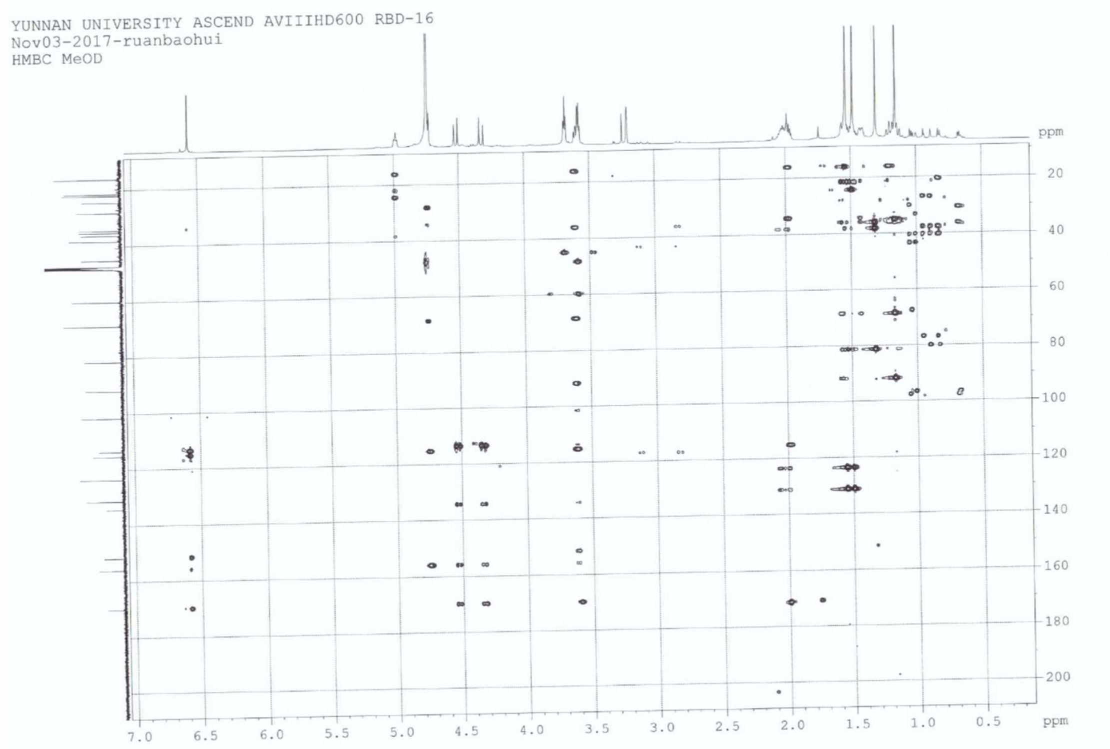

Figure S6. HMBC spectrum of compound **1** in MeOD (600 MHz)

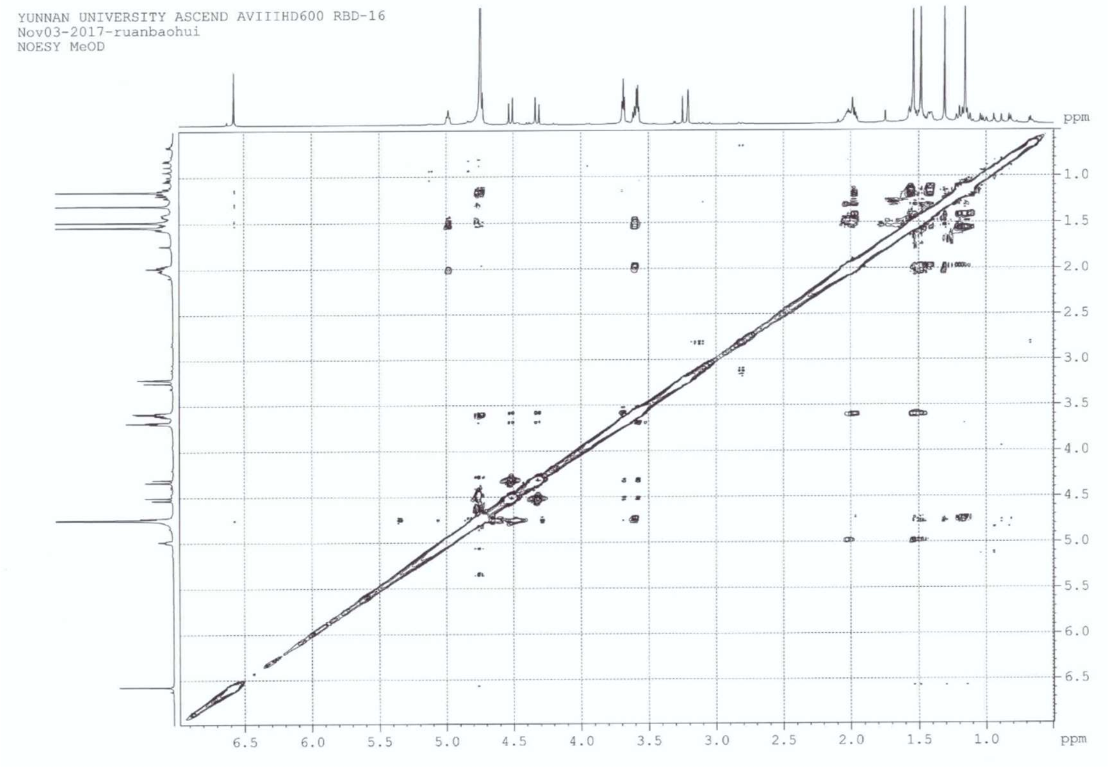

Figure S7. ROESY spectrum of compound **1** in MeOD (600 MHz)

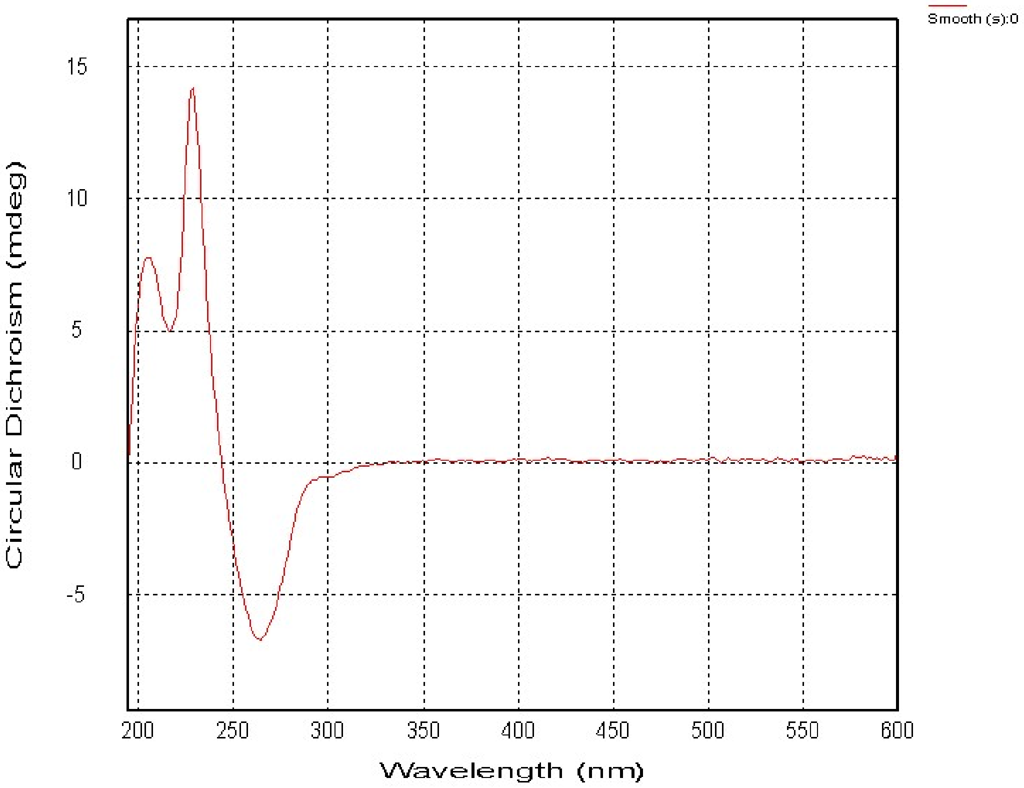

Figure S8. CD spectrum of compound **1**

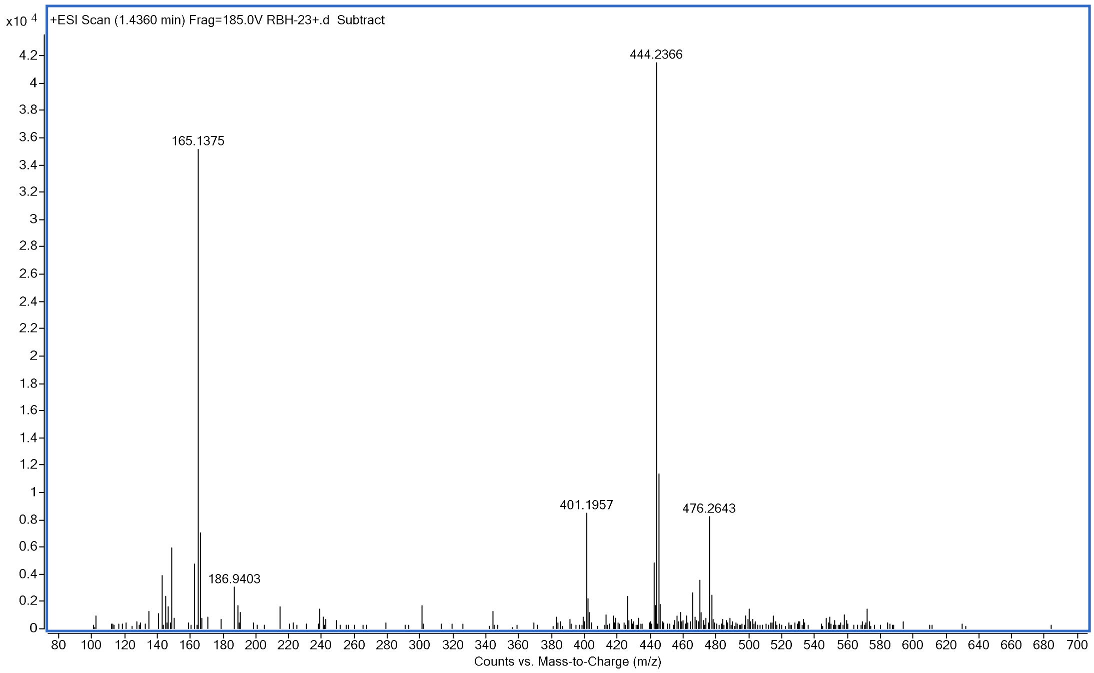

Figure S9. HRESIMS spectrum of compound **2**

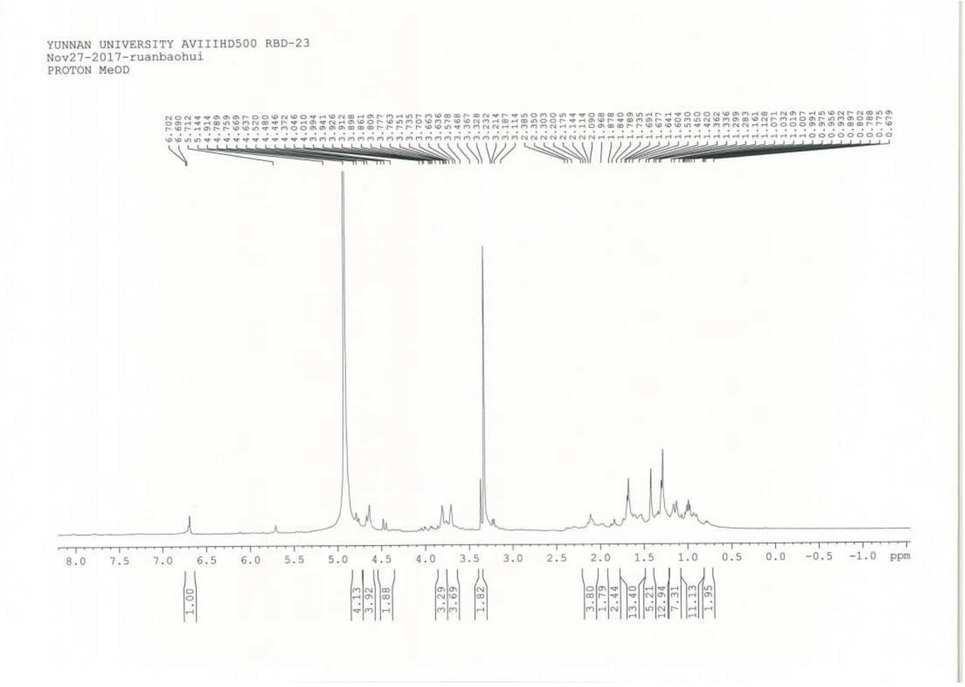

Figure S10. ^{1}H NMR spectrum of compound **2** in MeOD (500 MHz)

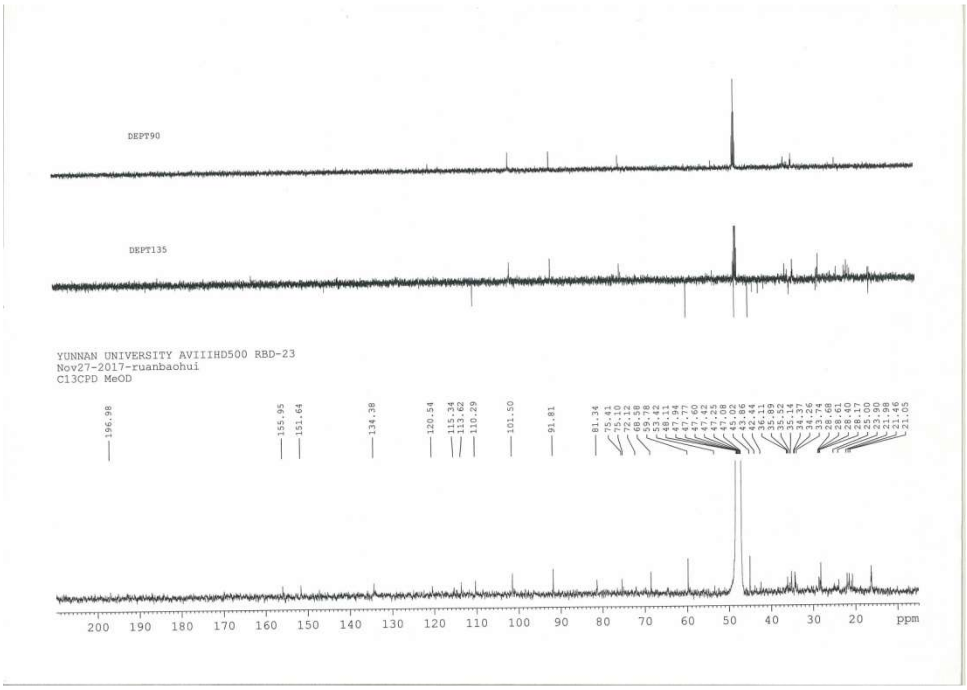

Figure S11. ^{13}C NMR spectrum of compound **2** in MeOD (125 MHz)

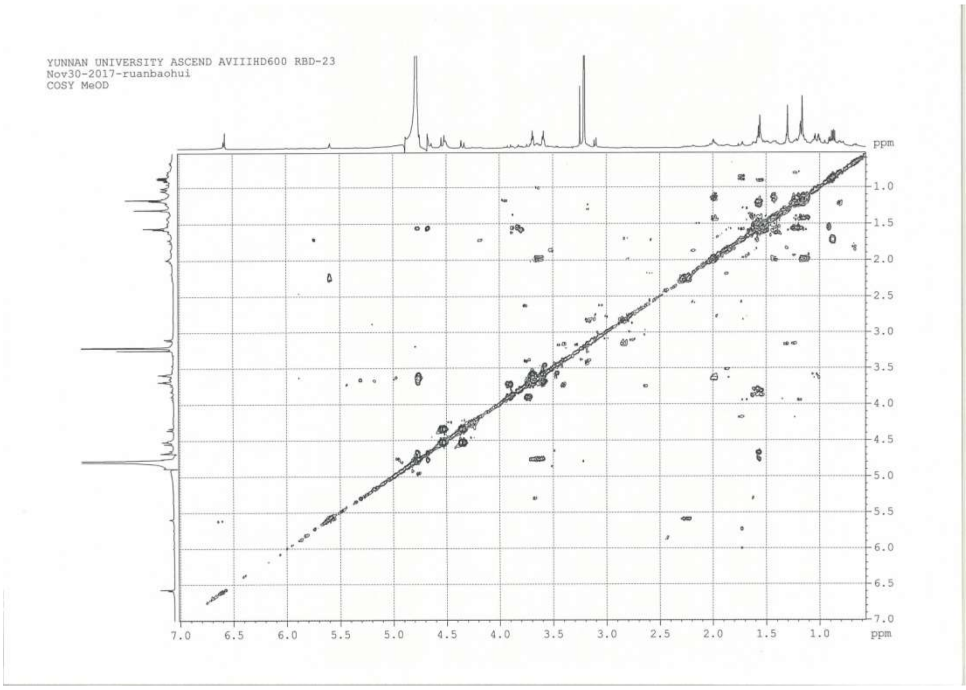

Figure S12. COSY spectrum of compound **2** in MeOD (600 MHz)

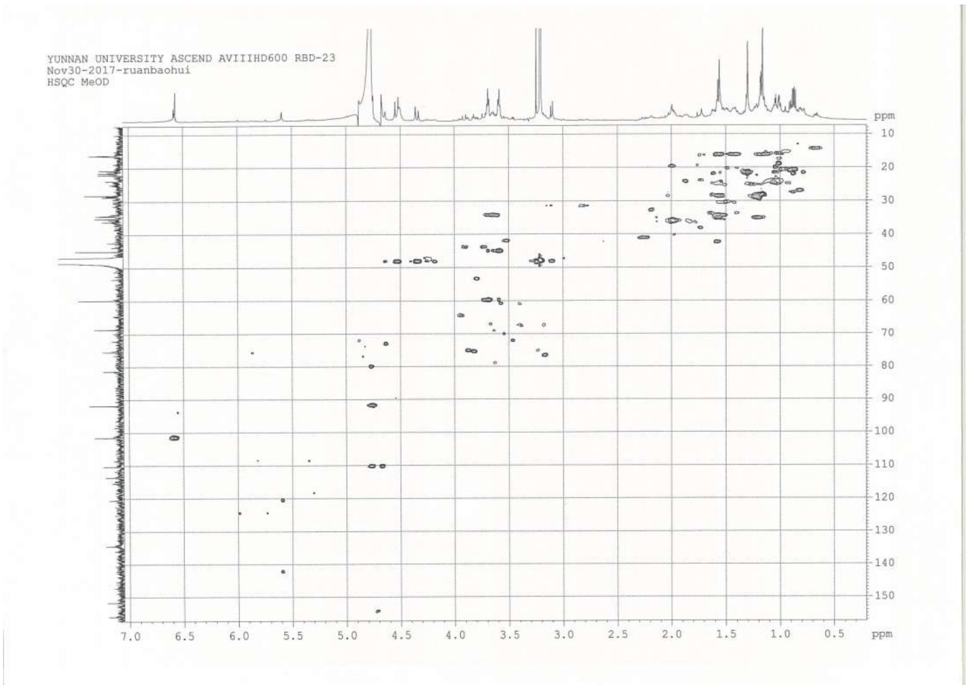

Figure S13. HSQC spectrum of compound **2** in MeOD (600 MHz)

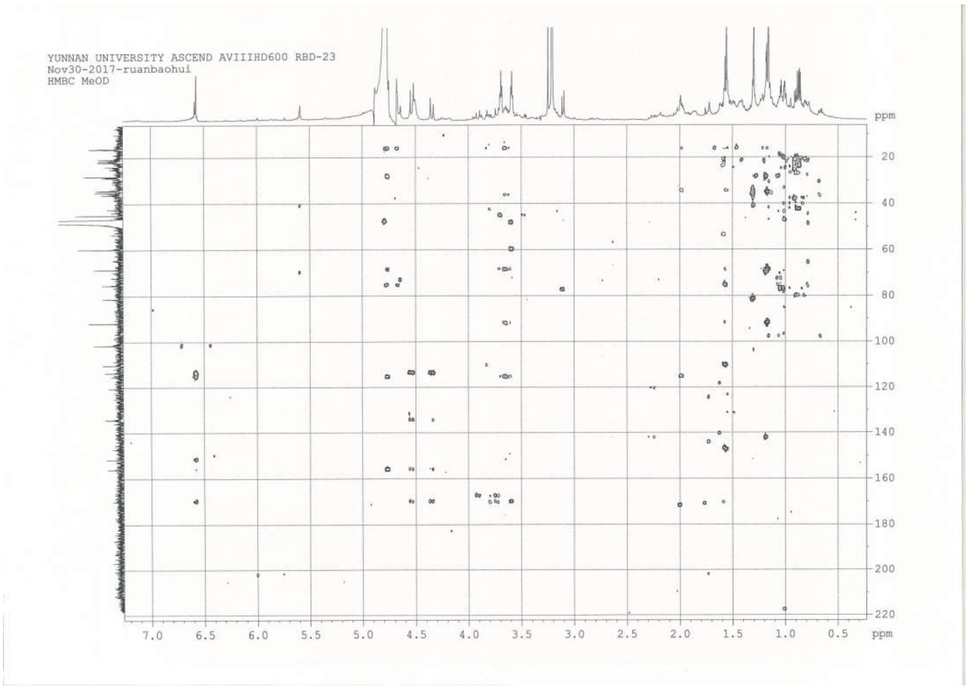

Figure S14. HMBC spectrum of compound **2** in MeOD(600 MHz)

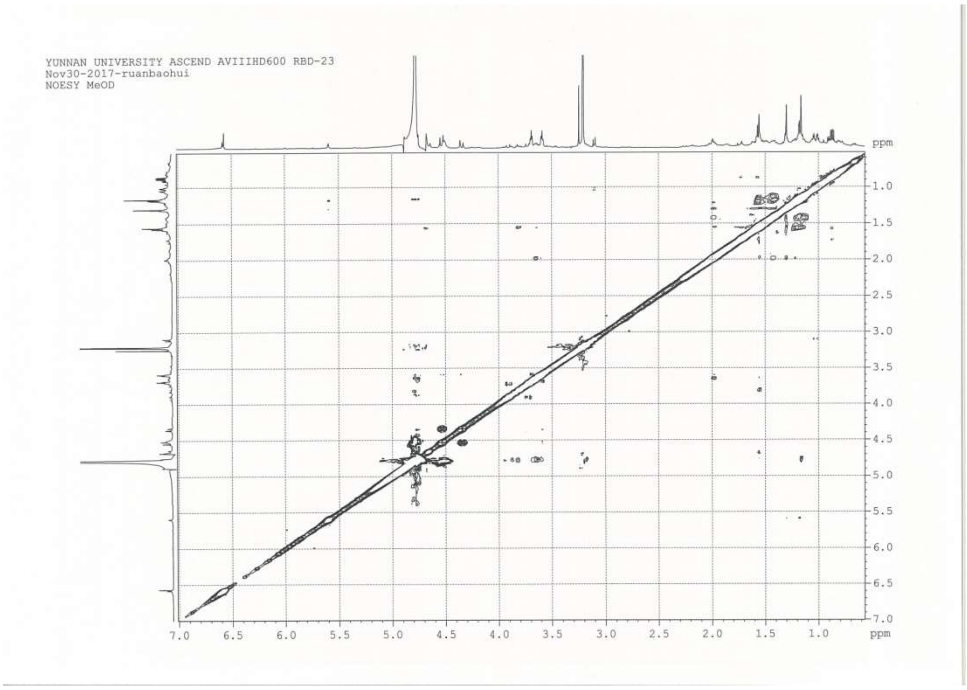

Figure S15. ROESY spectrum of compound **2** in MeOD (600 MHz)

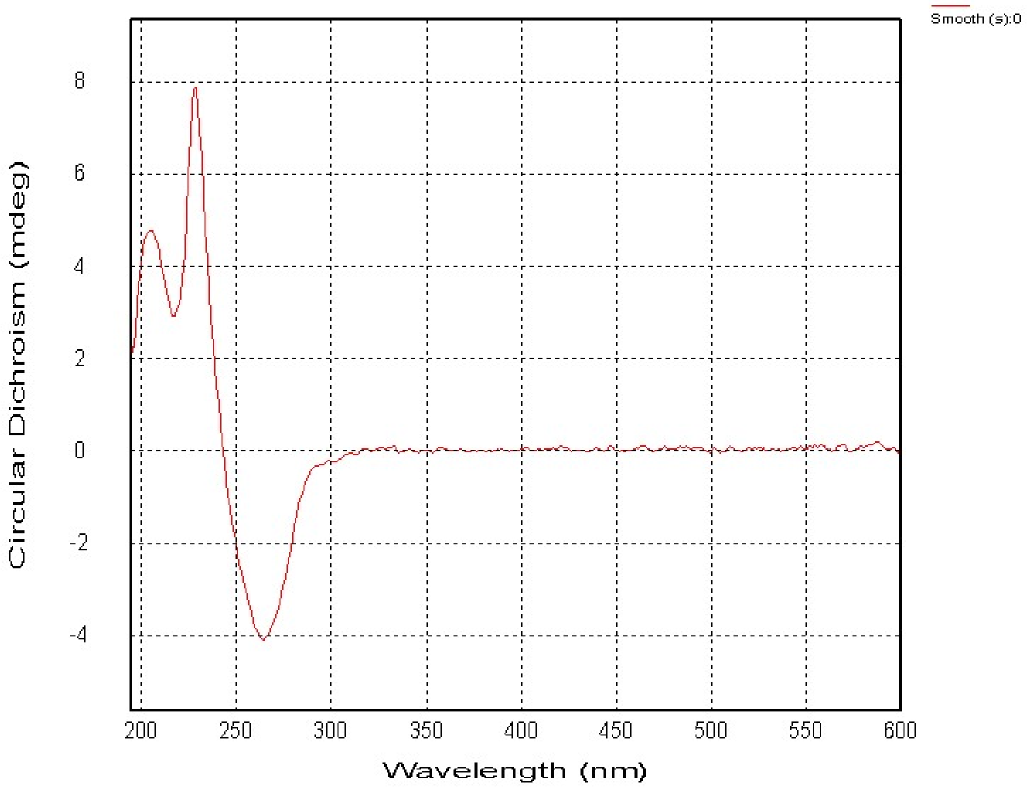

Figure S16. CD spectrum of compound **2**
